# Dynamics of Vegetation and Soil in Long‐Term Artificial Sand Control Projects in the Ulan Buh Desert

**DOI:** 10.1002/ece3.73296

**Published:** 2026-03-15

**Authors:** Benmo Li, Dong Wang, Yujie Xue, Runze Liu, Jingwen Li

**Affiliations:** ^1^ School of Ecology and Nature Conservation Beijing Forestry University Beijing China; ^2^ State Key Laboratory of Efficient Production of Forest Resources Beijing China

**Keywords:** desertification, ecological restoration, sand control projects, Ulan Buh Desert, vegetation succession

## Abstract

Desertification is a severe ecological problem worldwide under global change. China has devoted extensive and effective efforts to desertification prevention and sand control. However, the long‐term effectiveness of artificial sand control, particularly its impact on vegetation and soil evolution, remains understudied. To clarify the effect of different years of restoration on plant community characteristics and soil properties, and the overall succession trend of vegetation and soil during long‐term restoration, this study carried out a field investigation in the straw checkerboard barriers under different years of restoration (0 year: unrestored stage; 1, 5 years: initial stage; 10, 15 years: late stage) in the Ulan Buh Desert. Comprehensive evaluation models were used to quantify vegetation and soil improvement under restoration efforts, and a vegetation‐soil coupling coordination degree model was employed to explore their coordination level over different restoration periods. Results showed significant differences in plant species diversity among restoration years (*p* < 0.05). Plant diversity indices and coverage in the late stage of restoration were significantly higher than those in the unrestored and initial stages. After 10 years of restoration, the indicator species of sand fixation *Artemisia* *desertorum* reached maturity, relatively stable near‐natural communities. Meanwhile, leaf and root nutrient contents had inflection points in this stage. Soil properties varied significantly across restoration years (*p* < 0.05). The comprehensive evaluation indices of vegetation growth and soil quality peaked around the 10th year of restoration and then tended to stabilize. The coupling coordination degree between vegetation and soil was 0.63 in the 10th year of restoration, suggesting that the vegetation‐soil system was in a state of well‐coordinated development. Taken together, vegetation and soil succession tended to stabilize after 10 years of artificial sand control. These results will provide a theoretical basis for sand control projects, especially for the late‐stage management of the projects.

## Graphical abstract

This study investigates the long‐term synergistic evolution of vegetation and soil in artificial sand‐control projects (using straw checkerboard barriers) in China's Ulan Buh Desert. By analyzing areas with different restoration times (0–15 years), it identifies ~10 years as a critical inflection point where plant communities mature into stable, near‐natural states and soil quality significantly improves, leading to a well‐coordinated and benign development vegetation‐soil system. The research provides a vital scientific basis for managing the late stages of desertification control projects.

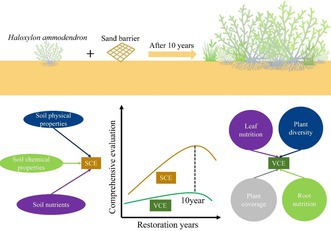

**Abbreviations**
AN: available nitrogen
AP: available phosphorus
BD: bulk density
EC: electrical conductivity
NN: nitrate nitrogen
SCE: soil comprehensive evaluation
SOC: soil organic carbon
SWC: soil water content
TN: total nitrogen
TP: total phosphorus
VC: vegetation coverage
VCE: vegetation comprehensive evaluation

## 1 Introduction

Desertification control, as a central issue related to the stability of dryland ecosystems under global change, is directly associated with the sustainable development of nearly 3 billion human beings (Zhang et al. 2023). Desertified areas are one of the most ecologically vulnerable areas on the planet and are highly prone to degradation under external disturbance. Currently, the problems of water imbalance, biodiversity loss, and landscape fragmentation in arid and semi‐arid regions have been intensified by the synergistic effect of climate change and anthropogenic activities, becoming major challenges in ecology that need urgent solutions (Dong et al. 2014). The Ulan Buh Desert in northwest China, as a typical representative of temperate deserts worldwide, is faced with accelerated desertification processes which not only result in sharp declines in land productivity in this region but also transport a large amount of sand to the upper reaches of the Yellow River, posing a unique threat to the ecological security of the river basin (G. Li et al. 2015). China has carried out several large‐scale vegetation restoration projects in the Ulan Buh Desert since 1978, such as the Three‐North Shelterbelt Project, which has significantly curbed the expansion of desertification and promoted the recovery of ecosystem functions (Deng et al. 2019). Specifically, the core technique “straw checkerboard barriers + artificial vegetation” has managed to accelerate plant community succession from “mobile dune communities” to “fixed/semi‐fixed dune communities” by altering microhabitats and species composition in the desert ecosystem (Kou and Jiao 2022; Lu et al. 2023). This typical engineering‐biological combined sand fixation technology forms a vital technical complement to the innovative sand solidification methods such as enzyme‐catalyzed mineralization and biomineralization (Miao et al. 2020, 2024), which are recognized as promising approaches for desertification control due to their wide adaptability and ecological restoration without irrigation (Miao 2025). However, existing studies mostly focus on short‐term (< 10 years) shifts in plant community structure, and there is a knowledge gap regarding the synergistic mechanism of vegetation‐soil system functions on a long time scale (> 10 years). For instance, the threshold for the dynamic coupling between vegetation succession and soil physicochemical properties has not been clarified, and there is a lack of quantitative analysis on the driving mechanism of the “inflection point” of ecological restoration (such as root‐soil feedback in dominant species). Such knowledge gap greatly hinders the scientific evaluation of the long‐term effectiveness of sand control projects in arid areas (Kou and Jiao 2022; Lu et al. 2023). Therefore, revealing general laws in the restoration of vegetation‐soil system in the Ulan Buh Desert based on long‐time series data will not only meet the core requirement of the sustainability evaluation of regional ecological projects but also provide case support for threshold identification and management optimization in the restoration of global dryland ecosystems.

The coordination between vegetation and soil is beneficial for the sustainable development of land use (Z. Li et al. 2022; Maxwell et al. 2018; Yu et al. 2022). Vegetation restoration alters regional coverage patterns and conditions of the underlying surface, and favorable soil conditions in turn act as key drivers of the sustainable development of vegetation (Alderson et al. 2025; Cui et al. 2020). Soil nutrient content and its spatial distribution patterns significantly affect plant growth, development, reproduction, and succession (Gou et al. 2019), and vice versa. Soil moisture is a critical factor that regulates vegetation growth and development (Lian et al. 2021). Water deficiency may lead to plant death, thereby affecting the spatial distribution of vegetation (McKiernan et al. 2017). Rainfall is the primary limiting factor of plant growth in the Ulan Buh Desert, China (Feng et al. 2024). In the late stage of vegetation restoration, vegetation development can improve soil quality (Long et al. 2024). Some studies have demonstrated that vegetation improves soil hydrological properties by altering soil physical properties. Meanwhile, different vegetation types and restoration methods would exert varying effects on soil properties (S. Ma et al. 2023; Tang et al. 2024). For instance, different vegetation restoration methods are found to significantly increase soil water‐holding capacity, saturated hydraulic conductivity, aggregates, and organic matter (Dou et al. 2020; He et al. 2021; Hao et al. 2024). Taken together, previous studies on vegetation restoration have mostly focused on the impacts of restoration methods and vegetation types on soil properties, as well as the interactions between vegetation and soil (Hao et al. 2024; He et al. 2021; L. Ma et al. 2024; Yan et al. 2024), while comparative studies on the long‐term dynamics of ecological restoration under consistent management remain relatively scarce. The Ulan Buh Desert is a major source of wind‐blown sand in China, characterized by climate aridity, water scarcity, barren soil, and severe secondary salinization. It has long been ecologically restored using straw checkerboard barriers and artificial *Haloxylon ammodendron* forests. Since different vegetation restoration durations yield varying effects on arid ecosystems, selecting an appropriate duration for ecological restoration is of great importance for desert control in the Ulan Buh Desert. A comprehensive evaluation model is a useful tool for quantitatively evaluating multiple factors, which can combine different indices to generate an integrated evaluation result. It has been widely applied in the effectiveness evaluation of ecological restoration (Guo et al. 2021; Feng et al. 2023). Thus, this study used comprehensive evaluation models to quantify vegetation and soil improvement under restoration efforts.

Coupling is a classical concept in physics that describes the process in which two or more independent systems interact to produce matter or energy (Y. Lu et al. 2019). Currently, numerous scholars have developed coupling coordination degree models for vegetation and soil to quantitatively evaluate their interactions, which can help to comprehensively understand the coordination of vegetation and soil in the process of ecological restoration (Gao et al. 2022). The coupling coordination degree between vegetation and soil increases and then decreases with increasing years of restoration (Gao et al. 2022; Tang et al. 2022). Soil factors play a more dominant role in driving the restoration process, compared to vegetation characteristics (X. Gao et al. 2019; Qiu et al. 2025; Yang et al. 2023; Zhong et al. 2022). In addition, high water‐use efficiency of desert vegetation biomass and its delayed response to soil moisture shifts highlight the complexity of the coupling processes between vegetation and soil in arid regions (Wang et al. 2017). The coupling relationship between vegetation and soil is jointed determined by multiple factors, such as climate, landform, etc. Its spatial heterogeneity requires that restoration strategies should adapt to local conditions. The special coupling mechanism in arid regions not only expands the application boundary of traditional coupling theory, but also provides a critical scientific basis for desert ecological restoration under global change.

This study investigated straw checkerboard barriers with similar topographic conditions but under different years of restoration, aiming to (1) explore the effects of different years of restoration on plant community characteristics and soil physicochemical properties, and the overall succession trend of the vegetation‐soil system under long‐term restoration efforts; (2) quantify vegetation and soil improvement using a comprehensive evaluation model, and identify the time threshold for ecological restoration; (3) determine the level of coordination between vegetation and soil under different years of restoration with a vegetation‐soil coupling coordination degree model.

## 2 Materials and Methods

### 2.1 Study Area

**FIGURE 1:**
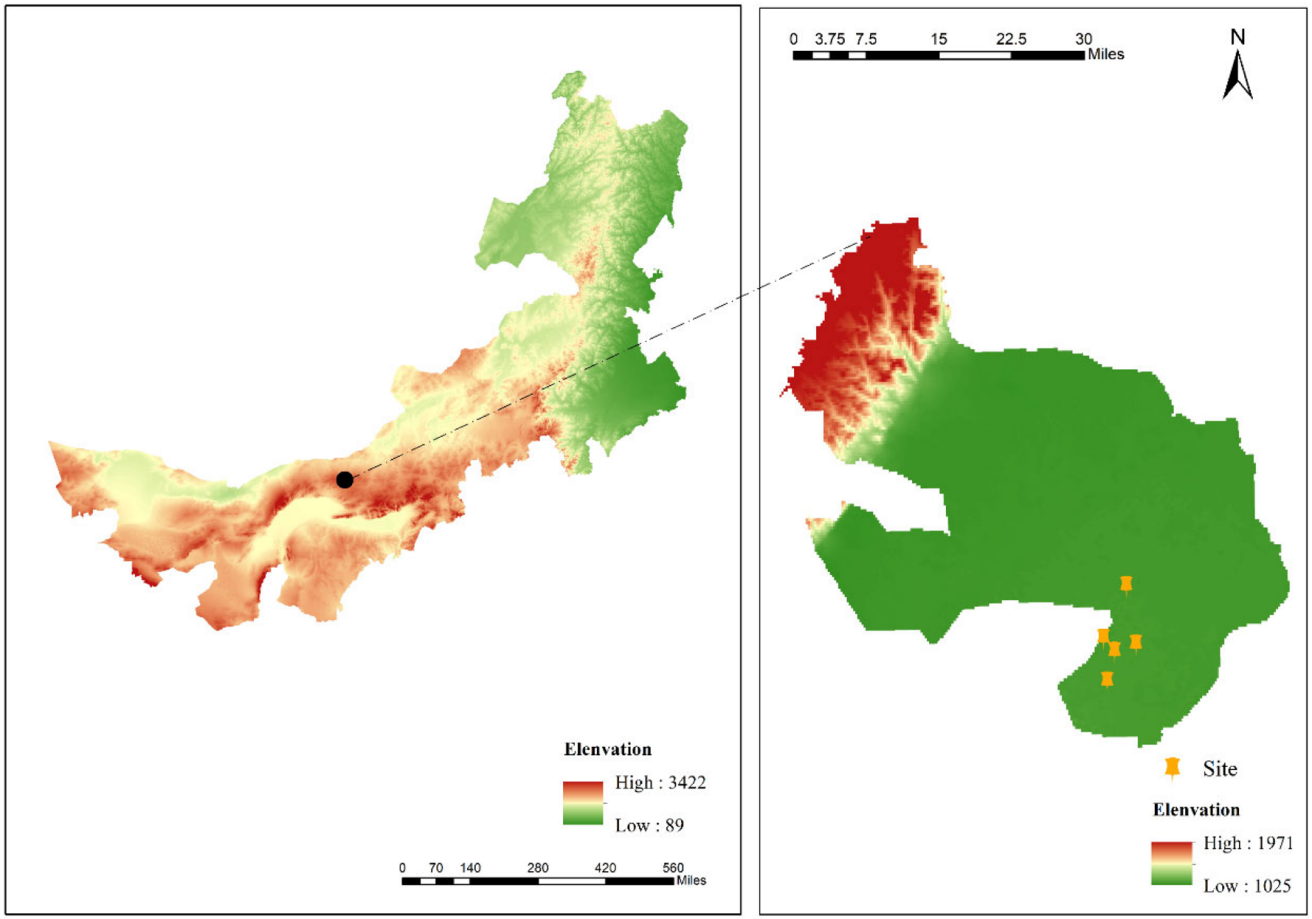
Overview of the study area.

The study area is located in the northeastern part of the Ulan Buh Desert (40°10′54″‐40°16′32″ N, 106°41′18″‐106°54′30″ E), and the transition zone between the Helan Mountains and the Hetao Plain in Inner Mongolia, China, with a total area of 37.58 km^2^ (Figure 1). It is adjacent to the Nailun Lake National Wetland Park, with the Yellow River floodplain located on its southwest side. The landform is characterized by crescent‐shaped mobile dune chains (> 60%), saline‐alkali depressions (25%), and fixed dune patches (15%). The overall terrain slopes gently from southwest to northeast, with an elevation of 1054–1106 m (Table 1). The study area has a cold desert climate (Köppen climate classification), with an average annual temperature of 7.8°C (lowest temperature: −29.6°C, highest temperature: 39°C), an average annual precipitation of 142.7 mm (67% concentrated in June–August), and a potential evaporation of 2258.8 mm. West wind prevails in this area (3.7 m/s on average), shaping the steep morphology of dune leeward slopes (> 30 degrees). The soil is dominated by eolian sandy soil, with a sand content of > 85% and a pH of 8.2–8.6. The groundwater depth is 1.5–3.0 m. The natural vegetation is dominated by *Artemisia saxifraga*, *Sargassum*, and reed communities, and vegetation coverage in mobile dune areas is < 8%.

**TABLE 1:** Basic information of sampling sites.

Restoration years	Longitude	Latitude	Altitude	Number of species
0 year	106.86	40.37	1051	2
1 year	106.83	40.26	1060	4
5 years	106.82	40.28	1055	4
10 years	106.81	40.22	1068	11
15 years	106.88	40.27	1055	10

Since 2005, the Ulan Buh Desert has been subject to annual ecological restoration and desertification control projects. Prior to the sand fixation measure implementation, the unrestored land was first leveled, after which 1 × 1 m straw checkerboard sand barriers were laid out in a standard grid pattern, and artificial *Haloxylon* *ammodendron* seedlings were planted at the grid centers with a density of 800–1200 plants/ha. This integrated engineering measure has achieved stable sand fixation and vegetation colonization, and the vegetation coverage in this area has subsequently been enhanced to 28%–35%, rendering the Ulan Buh Desert a typical desertification control area in the arid regions of northwest China.

### 2.2 Soil Sampling and Analysis

Considering that the same ecological restoration measures have been continuously implemented in this area for a long time, and the desert ecosystem has shown long‐term stable soil, water, and climate conditions. This study adopted a space‐for‐time substitution approach. Field sampling and investigation were conducted in the Ulan Buh Desert from July to August 2024. Specifically, areas with different restoration years (0, 1, 5, 10, and 15 years) were selected as the research objects, where the 0‐year area was an unrestored area serving as the control, and the 1 year, 5 year, 10 year, and 15 year restoration areas corresponded to the restoration projects established in 2023, 2019, 2014, and 2009 respectively.

**FIGURE 2:**
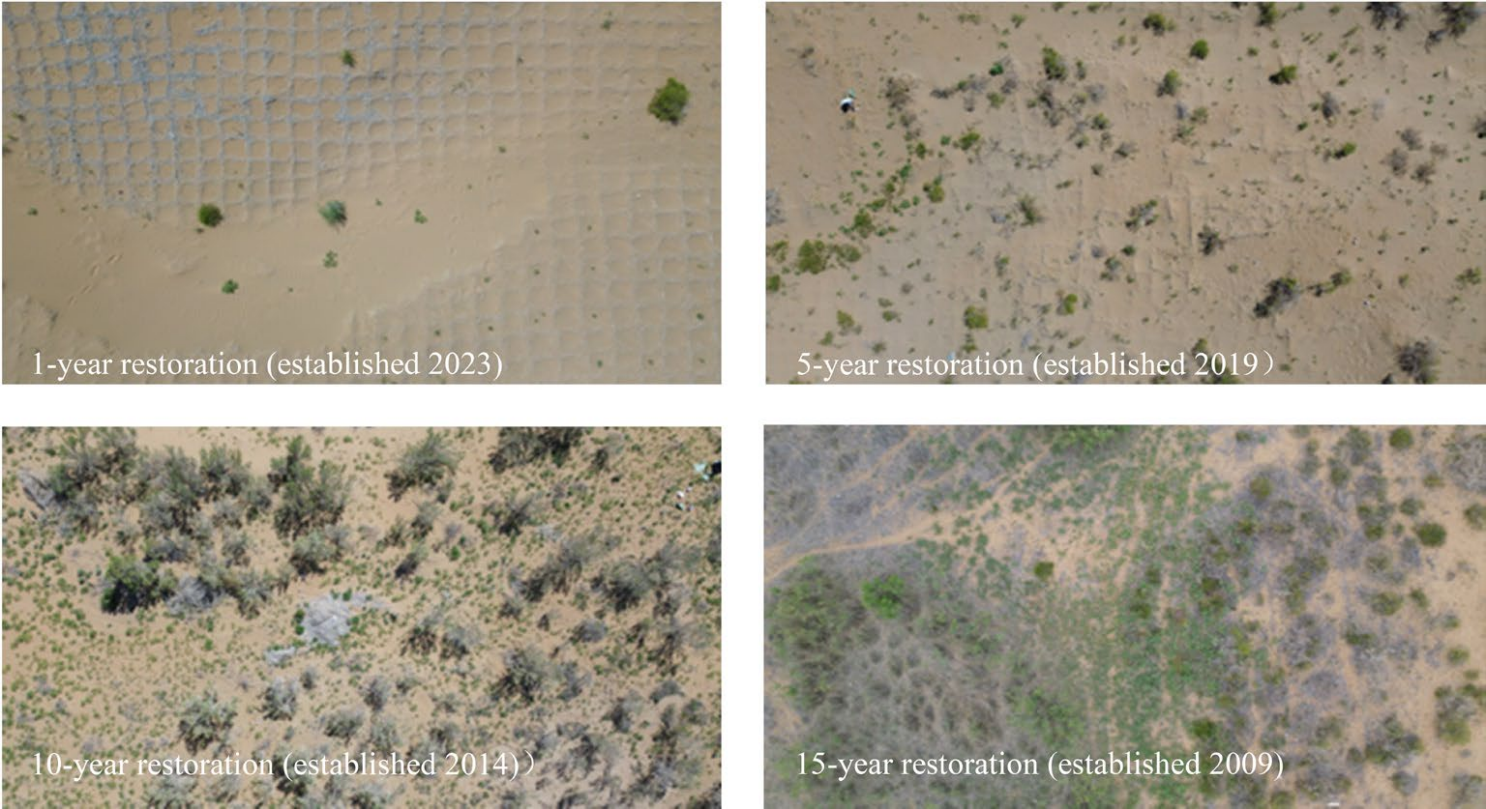
Vegetation and soil changes under different years of restoration (clear age annotations below each panel confirm the consistency and succession stages of the study sites). Top left: 1 year restoration (established 2023); Top right: 5 year restoration (established 2019); Bottom left: 10 year restoration (established 2014); Bottom right: 15 year restoration (established 2009).

All sampling plots were set in the same straw checkerboard barrier project with identical sand fixation techniques, and different restoration years represented the ecological succession stages of the desert ecosystem. The specific distribution of the study area is shown in Figure 1, and the vegetation communities and soil changes under different restoration years are shown in Figure 2. Figure 2 shows the vegetation and soil changes under different restoration years in the same sand control project of the Ulan Buh Desert, with clear age annotations below each panel confirming the consistency and succession stages of the study sites.

For each restoration period, five plots (20 × 20 m) were randomly established. In each subplot, soil at the depth of 0–20 cm (surface layer), 20–40 cm (subsurface layer), and 40–60 cm (deep layer) was systematically collected with a soil auger (5 cm in diameter) following the nine‐point grid method (3 × 3). Soil samples from the same depth were mixed into 75 composite samples (5 plots × 3 depths × 5 ages). Fresh soil samples were immediately sealed in airtight containers. Gravimetric water content was determined by drying samples in an oven at 105°C for 24 h (Bao 2010), while bulk density was measured using the core method (Blake and Hartge 1986). Air‐dried soil samples were sieved through 2 mm and 0.15 mm sieves for the following analyses:

#### 2.2.1 Soil Organic Carbon (SOC)

Determined by the Walkley–Black wet oxidation method (Nelson and Sommers 1996). A 0.5 g soil sample was mixed with 10 mL of 0.167 M potassium dichromate (K_2_Cr_2_O_7_) and 20 mL of concentrated sulfuric acid (H_2_SO_4_) for digestion, followed by titration with 0.5 M ferrous ammonium sulfate (FeSO_4_·(NH_4_)_2_SO_4_).

#### 2.2.2 Soil Total Nitrogen (TN)

Measured using the Kjeldahl method coupled with a SEAL AA3 analyzer (Bremner 1960). A 1 g soil sample was digested with 10 mL of H_2_SO_4_ and 1.85 g of catalyst (K_2_SO_4_:CuSO_4_:Se = 10:1:0.1) at 380°C for 120 min, then subjected to colorimetry with sodium salicylate‐sodium hypochlorite (660 nm, DIN 38406 Part 23).

#### 2.2.3 Soil Total Phosphorus (TP)

Determined by AA3 after HClO_4_‐H_2_SO_4_ digestion (Olsen and Sommers 1982). A 0.5 g soil sample was mixed with 5 mL of H_2_SO_4_ overnight, then 10 drops of HClO_4_ were added for digestion at 180°C until clear. Molybdenum blue colorimetry was used for quantification (880 nm, ISO/DIS 15681–2).

#### 2.2.4 Soil Available Phosphorus (AP)

Measured by the Olsen method (Olsen et al. 1954). A 5 g soil sample was shaken with 100 mL of 0.5 M NaHCO_3_ (pH 8.5) for 30 min, then analyzed by AA3 (880 nm, detection limit 0.031 mg/L, DIN/EN/ISO 15681‐2).

#### 2.2.5 Soil Ammonium Nitrogen (NH_4_^+^‐N) and Nitrate Nitrogen (NO_3_^−^‐N)

Extracted with 2 M KCl (soil‐to‐solution ratio 1:5) by shaking for 1 h, filtered through Whatman No. 42 filter paper, and quantified using a flow injection analyzer (FIAstar 5000; Foss Tecator).

#### 2.2.6 Particle Size Distribution

Organic matter was removed with 30% H_2_O_2_, and samples were dispersed with 0.5% sodium hexametaphosphate. Particle size was determined by laser diffraction (Malvern Mastersizer 3000) (Gee and Bauder 1986).

### 2.3 Vegetation Survey and Analysis

In each subplot, five 1 m × 1 m herbaceous sample plots were systematically set up. Within each sample plot, the species composition, mean height (cm), total coverage (%), and subspecies density (plants/m^2^) of all vascular plants were recorded in detail. Representative individuals of dominant plant species (*Haloxylon ammodendron*, *Artemisia desertorum*, *Phragmites australis* ) were selected for sampling. For each target species, root and leaf (or assimilated branch) samples were collected from three healthy mature individuals within each subplot. The collected plant tissue samples were immediately stored in an ice box. Upon return to the laboratory, the samples were rinsed three times with deionized water to remove surface adherents. Rinsed samples were placed in a freeze dryer and freeze‐dried at −55°C for 48 h. The freeze‐dried plant tissue samples were ground in a ball mill and passed through a 0.15 mm sieve for chemical analysis.

Total carbon (C) content was determined using the potassium dichromate oxidation‐external heating method (Nelson and Sommers 1996). Specifically, approximately 0.2 g of sample powder was accurately weighed, digested in a potassium dichromate‐sulfuric acid solution, and then titrated with a standard ferrous sulfate solution.

For total nitrogen (N) and total phosphorus (P) contents, an appropriate amount (approximately 0.1–0.2 g) of sample powder was accurately weighed. The test solution was prepared via the concentrated sulfuric acid‐hydrogen peroxide digestion method. The contents of nitrogen (usually in the form of ammonium nitrogen) and phosphorus (usually in the form of orthophosphate) in the digested solution were determined using a continuous flow analyzer (Auto Analyzer 3, SEAL Analytical or equivalent models). The specific determination methods followed the instrument‐matched indophenol blue method and molybdenum–antimony–ascorbic acid colorimetric method, respectively.

### 2.4 Data Analysis

All statistical analyses in this study were conducted using R software (version 4.4.2). A two‐way analysis of variance (two‐way ANOVA) was applied to examine the main effects of the fixed factors (restoration year, referred to as “Year”; and soil depth, referred to as “Depth”) and their interaction effect on each soil physical and chemical property indicator. If the interaction effect was significant (*p* < 0.05), indicating that the main effects depended on the levels of the other factor, simple effect analyses were performed. When the results of the simple effect analyses were significant (*p* < 0.05), Tukey's Honestly Significant Difference (HSD) test was further used for post hoc multiple comparisons within the levels of the respective factor (*p* < 0.05). If the interaction effect was nonsignificant (*p* ≥ 0.05) but a main effect was significant (*p* < 0.05), Tukey's HSD test was employed for post hoc multiple comparisons (*p* < 0.05). A one‐way analysis of variance (one‐way ANOVA) was used to test the main effect of the fixed factor (restoration year, “Year”) on each vegetation characteristic indicator. When the effect was significant (*p* < 0.05), Tukey's HSD test was similarly applied for post hoc multiple comparisons (*p* < 0.05). Pearson's correlation coefficient was used to assess the pairwise linear correlations among vegetation characteristic indicators, soil physical property indicators, comprehensive evaluation functions, and coupling degrees. The significance levels were set at *p* < 0.05 (significant) and *p* < 0.01 (highly significant). To further explore the coupling relationships and driving pathways between vegetation and soil systems, a Partial Least Squares Path Modeling (PLS‐PM) was constructed. This model integrated observed variables and latent variables (e.g., “Vegetation Status”, “Soil Quality”) reflected by the observed indicators (Table 2). Path relationships were established based on research hypotheses. Model fit was evaluated using indicators including the significance of path coefficients (*p* < 0.05), the R^2^ (coefficient of determination) of endogenous latent variables, and the Goodness‐of‐Fit (GOF) index.

**TABLE 2:** Calculation formula of comprehensive evaluation model and coupling coordination degree model.

Model indices		Calculation	References
Comprehensive evaluation model	VCE	$\mathrm{VCE}=\sum_{i=1}^{p}E_{i}C_{i}$ Where, VCE is the comprehensive evaluation function of vegetation. Ei represents the membership value of the i evaluation index of vegetation. Ci is the weight of the i evaluation index of vegetation	Zhao et al. 2022
	SCE	$\mathrm{SCE}=\sum_{i=1}^{n}E_{i}C_{i}$ Where, SCE is the comprehensive evaluation function of soil. Wi is the membership value of the i evaluation index of soil. Ni is the membership value of the i evaluation index of soil	Zhao et al. 2022
Coupling coordination degree model	C	$C=\sqrt{\frac{\mathrm{VCE}\cdot \mathrm{SCE}}{{\left(\mathrm{VCE}+\mathrm{SCE}\right)}^{2}}}$ Where, C represents the vegetation‐soil coupling degree.	Peng et al. 2011
	C_h_	$C_{h}=\frac{\mathrm{VCE}+\mathrm{SCE}}{2}$ Where, Ch is the comprehensive harmonic index of vegetation‐soil coupling.	
	C_d_	$C_{d}=\sqrt{C\cdot C_{h}}$ Where, Cd is the vegetation‐soil coupling coordination degree.	

## 3 Results

### 3.1 Plant Diversity and Community Dynamics

As indicated in Table 3, plant species composition exhibited distinct stage‐wise changes with the progress of restoration. During the initial stage, plant species composition was relatively simple. Specifically, shrub species number did not increase after the first year of restoration, and herbaceous species number remained unchanged. During the late stage, particularly after 10 years of restoration, significant changes were observed in shrub species composition, accompanied by an increase in herbaceous species number, which led to a substantial rise in the total species number.

**TABLE 3:** Plant diversity and coverage under different years of restoration.

Plant community characteristics	0 year	1 year	5 year	10 year	15 year
Shannon index	0.31 ± 0.11 c	0.31 ± 0.17 c	0.70 ± 0.07 b	1.35 ± 0.07 a	1.30 ± 0.07 a
Margalef index	0.18 ± 0.06 d	0.27 ± 0.11 d	0.52 ± 0.03 c	1.55 ± 0.04 a	1.19 ± 0.08 b
Simpson index	0.80 ± 0.008 a	0.83 ± 0.10 a	0.63 ± 0.04 b	0.25 ± 0.00 c	0.37 ± 0.01 c
Pielou index	0.44 ± 0.17 bc	0.31 ± 0.12 c	0.58 ± 0.06 b	0.85 ± 0.01 a	0.74 ± 0.02 a
Plant coverage	4.17 ± 0.91 e	8.5 ± 0.96 d	25.25 ± 1.66 c	44.55 ± 0.88 b	61.90 ± 0.93 a

*Note:* Different lowercase letters indicate significant differences at *P* < 0.05 among different restoration years.

As shown in Table 3, Shannon index, Margalef index, Simpson index, and Pielou index all exhibited significant differences among different years of restoration (*p* < 0.05). Shannon index in the late stage and initial stage ranged from 1.37 to 1.42 and from 0.14 to 0.77, respectively. Margalef index in the late stage and initial stage ranged from 1.11 to 1.59 and from 0.16 to 0.55, respectively. These results indicated that long‐term restoration enhanced plant diversity. On the contrary, Simpson index in the initial stage was significantly higher than that in the late stage, suggesting that dominant species were more prominent under short‐term restoration. Pielou index in the late stage ranged from 0.72 to 0.86, while that in the initial stage ranged from 0.19 to 0.64, implying that long‐term restoration caused smaller interspecific difference and higher community evenness. In addition, vegetation coverage increased significantly with the progress of restoration, and the coverage in the late stage was 1.76 to 14.84 times as great as that in the initial stage.

### 3.2 Temporal Changes in Plant Nutrients During Vegetation Restoration

Significant differences were observed in leaf and root nutrient contents across different years of restoration, including leaf organic carbon content, leaf total phosphorus content, root organic carbon content, root total nitrogen content, and root total phosphorus content (*p* < 0.05).

**FIGURE 3:**
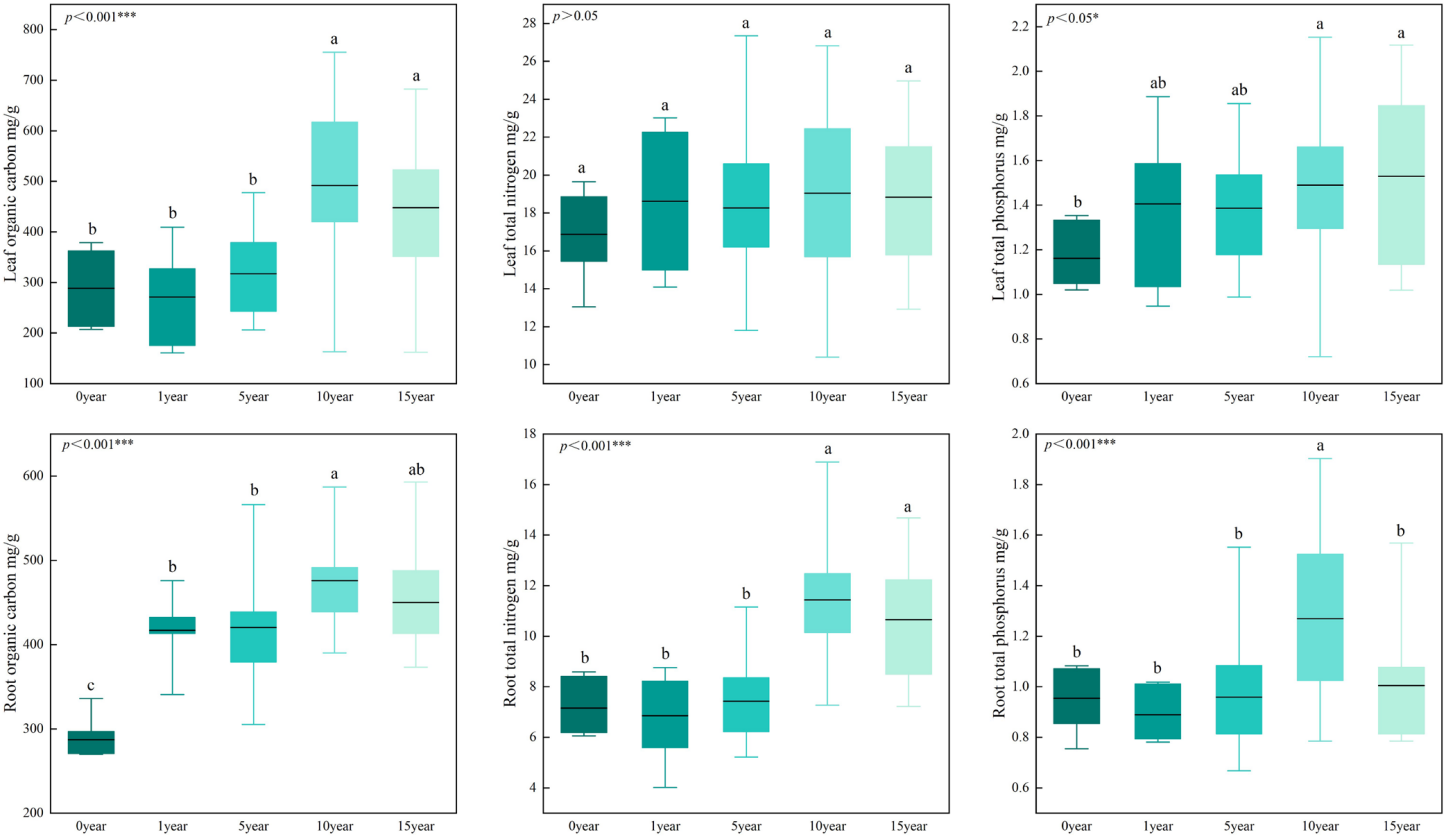
Leaf and root nutrient contents under different years of restoration. Different lowercase letters indicate significant differences at *p* < 0.05 among different restoration years.

Leaf organic carbon content in the late stage was significantly higher than that in the initial stage (*p* < 0.001), particularly reaching 491.91 ± 29.32 mg/g in the 10th year of restoration (Figure 3). There was no significant difference in leaf organic carbon content between the unrestored stage and initial stage. In addition, leaf organic carbon content decreased and then increased in the initial stage, and tended to stabilize in the late stage. As indicated in Figure 3, Leaf total nitrogen content did not exhibit significant differences across different years of restoration, but fluctuated greatly in the initial stage. Similarly, it reached a peak in the 10th year of restoration and then tended to stabilize. Leaf total phosphorus content in the unrestored stage was significantly lower than that in the late stage, and increased with the progress of restoration, reaching 1.53 ± 0.09 mg/g in the 15th year of restoration.

Root organic carbon content reached a peak of 475.88 ± 5.58 mg/g in the 10th year of restoration, which was significantly higher than that in the unrestored and initial stages (*p* < 0.001). Root organic carbon content in the initial stage was significantly higher than that in the unrestored stage but remained fluctuating in the initial stage and gradually stabilized in the late stage. Root total nitrogen content in the late stage was significantly higher than that in the unrestored stage and initial stage (*p* < 0.001). Root total nitrogen content did not display any significant difference between the unrestored stage and initial stage, and it initially decreased and then increased, reaching a peak in the late stage. Root total phosphorus content showed a unimodal pattern with the progress of restoration, reaching 1.27 ± 0.06 g/kg in the 10th year of restoration, which was significantly higher than that in other periods (*p* < 0.001).

### 3.3 Basic Soil Physicochemical Properties

**FIGURE 4:**
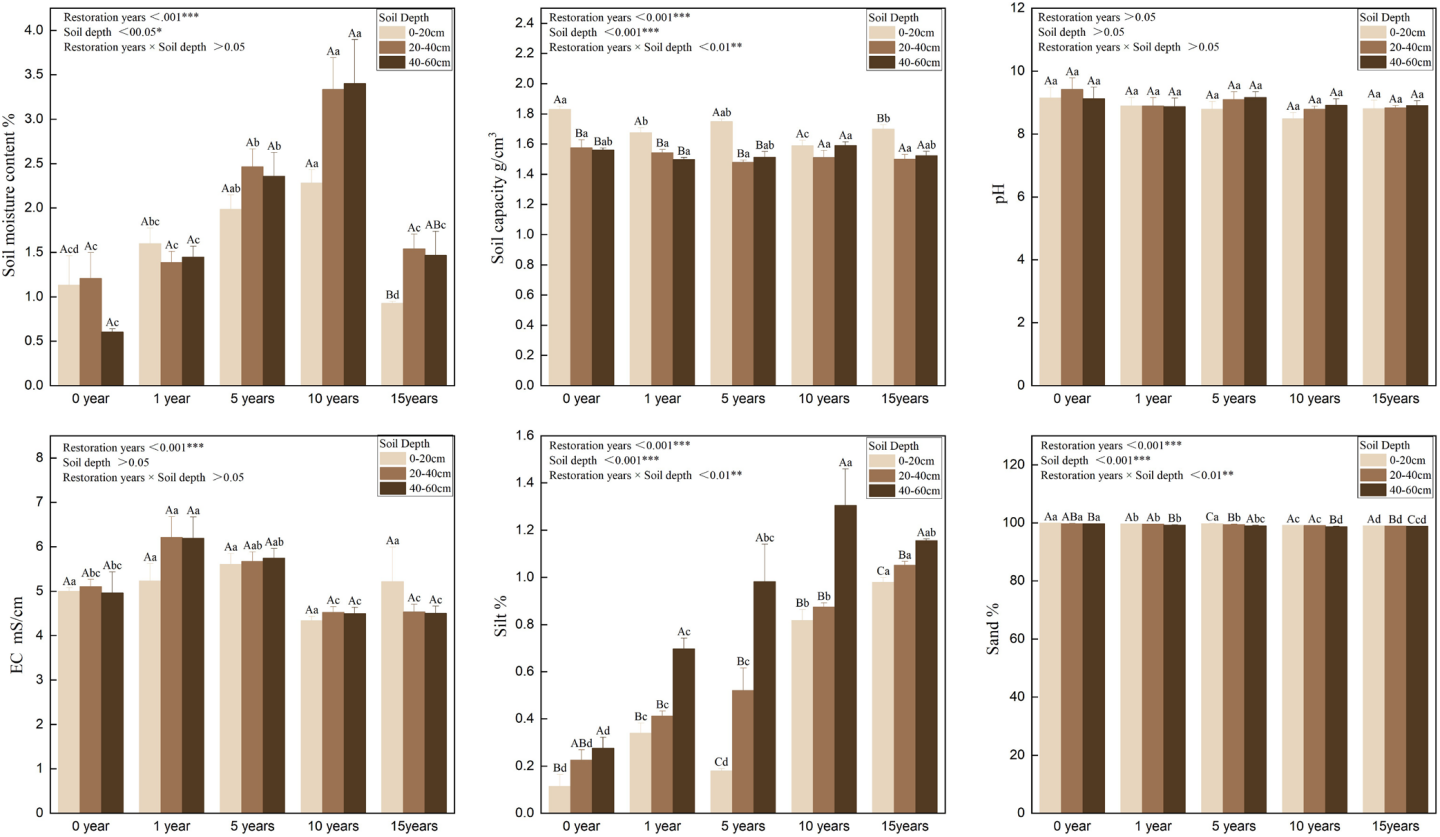
Soil physical properties under different years of restoration.

Soil physical properties differed significantly among different years of restoration (*p* < 0.05) (Figure 4). Soil moisture content of deep soil layers was significantly higher than that of surface soil layers in the 15th year of restoration. Soil moisture content in the 10th year of restoration was significantly higher than that in other periods (*p* < 0.05). Soil bulk density significantly decreased with increasing soil depth (*p* < 0.01) in all restoration periods. Soil bulk density of surface soil layers decreased significantly as restoration progressed (*p* < 0.05), and stabilised in the late stage of restoration. Soil pH showed no significant differences across different years of restoration or soil layers, but gradually decreased as restoration progressed. Soil EC exhibited no significant differences across soil layers, but differed significantly among different years of restoration (*p* < 0.001). Furthermore, soil EC in the late stage was significantly lower than that in the unrestored stage and initial stage. As indicated in Figure 4, the soil mechanical composition of each layer in all restoration periods followed the trend of sand > silt, without clay. As restoration progressed, soil silt content increased significantly while sand content decreased significantly. The silt contents of 0–20, 20–40, and 40–60 cm layers in the 15th year of restoration were 0.98%, 1.05%, and 1.16%, respectively, which were significantly higher than those in the unrestored stage. The sand contents of 0–20, 20–40, and 40–60 cm layers in the unrestored stage were 99.89%, 99.77%, and 99.72%, respectively, which were significantly higher than those in the 15th year of restoration. The observed changes in soil physical properties directly indicate an enhancement in the long‐term water‐holding capacity of the sand‐fixing area. The significant increase in soil silt content and reduction in sand content, combined with the decreased soil bulk density, optimiZed the soil pore structure, thereby improving the soil's ability to adsorb and retain water. This is further supported by the significantly higher soil moisture content observed in the 10th year of restoration, which suggests that the soil water‐holding potential reached a relatively stable and favorable state after 10 years of restoration, providing a critical water guarantee for the sustainable development of vegetation in the later stages of ecological restoration.

**FIGURE 5:**
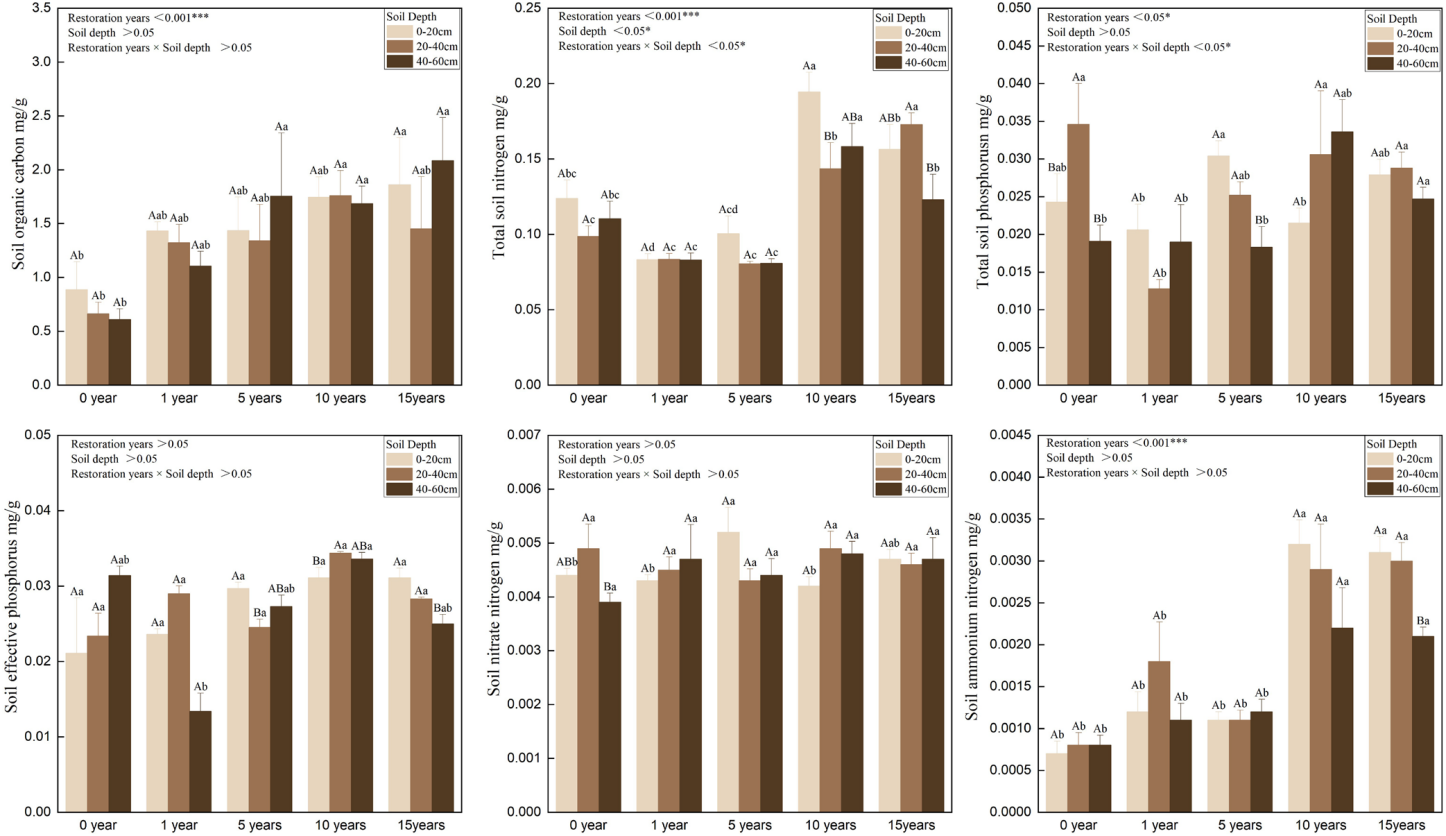
Soil chemical properties under different years of restoration.

As shown in Figure 5, soil chemical properties differed significantly across different years of restoration and soil depths (*p* < 0.05). Soil organic carbon (SOC) content had significant differences among different years of restoration (*p* < 0.001) and showed a fluctuating rise with the progress of restoration. SOC content of the surface and deep soil layers reached a peak of 0.45–3.09 mg/g and 1.22–3.56 mg/g in the 15th year of restoration, respectively. SOC content of the middle soil layer peaked at 1.21–2.48 mg/g in the 10th year of restoration. Total nitrogen (TN) content of the surface and deep soil layers reached the maximum in the 10th year of restoration, which was significantly higher than that in other restoration periods. TN content of the middle soil layer decreased and then increased as restoration progressed, peaking in the 15th year of restoration. The synergistic effect of restoration year and soil layer had a significant impact on TN content. Soil total phosphorus (TP) content differed significantly among different years of restoration. TP content of the surface and deep soil layers respectively peaked in the 5th and 10th year of restoration, while that of the middle soil layer peaked before the onset of restoration. Soil available phosphorus (AP) content and nitrate nitrogen (NN) content changed 0.02–0.04 mg/g and 0.004–0.005 mg/g on average across different years of restoration and soil depths, respectively, and thus no significant differences were observed. Soil available nitrogen (AN) content in the late stage of restoration was significantly higher than that in the initial stage and unrestored stage. Particularly, AN content of three soil layers all peaked after 10 years of restoration. Taken together, soil chemical properties were significantly improved and tended to stabilize after 10 years of restoration.

### 3.4 Vegetation‐Soil Coupling Relationship

**FIGURE 6:**
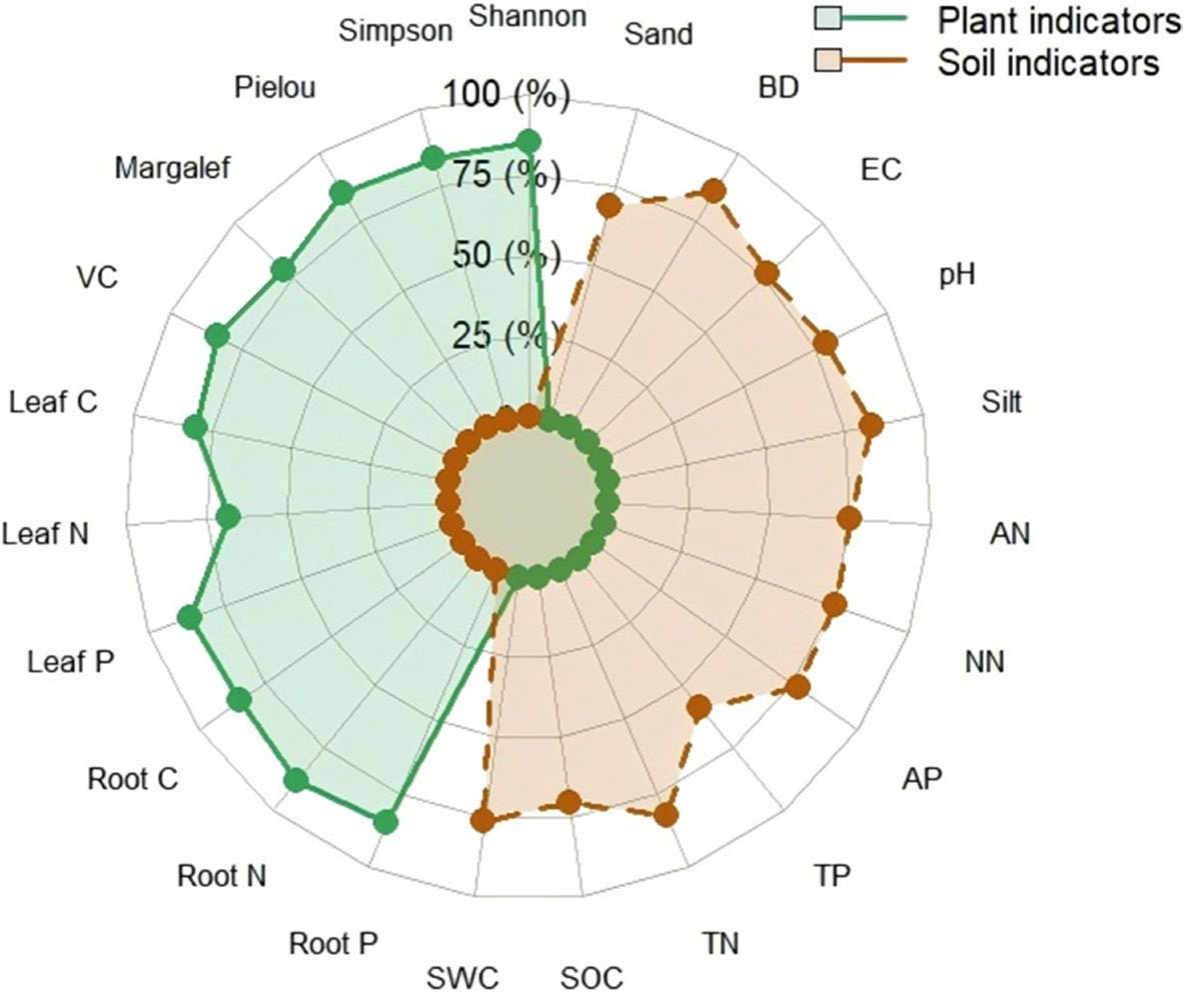
Vegetation and soil importance indices under different restoration years.

**FIGURE 7:**
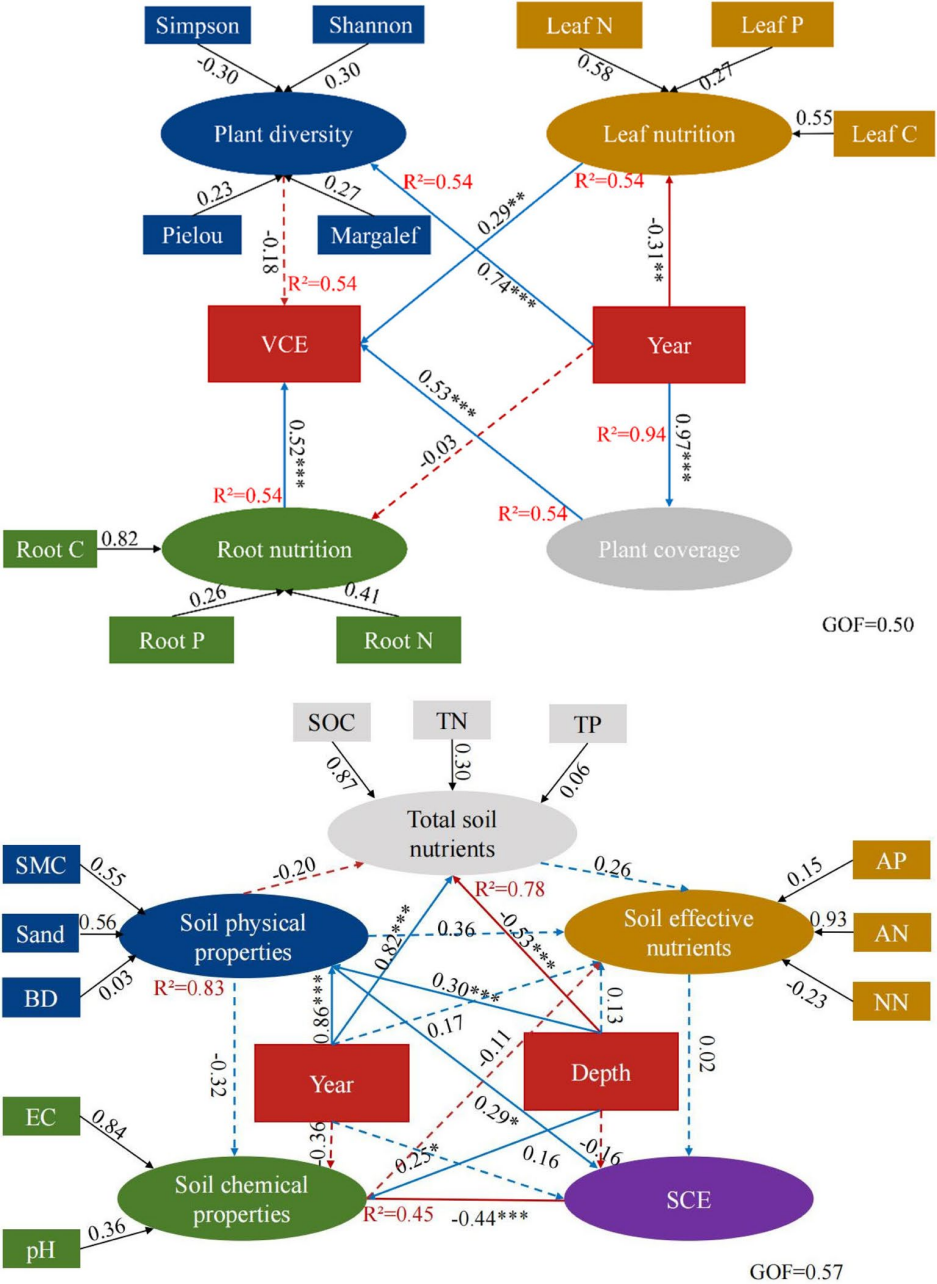
Partial least‐squares equation modeling. Solid lines indicate pathways that are significant at the 0.05 level of significance and dashed lines indicate pathways that are not significant. Asterisks following the pathway coefficients indicate the level of significance (*p < 0.05, **p < 0.01 and ***p < 0.001). The *R* ^2^ values attached to the factors represent the explanatory variables.

As shown in Figure 6, indicator weights showed a consistent trend with restoration years. Vegetation coverage, Shannon index, and root total nitrogen content played an important role in the comprehensive evaluation model for vegetation, while soil organic carbon content, soil moisture content, and soil total phosphorus content had a larger weight than other indices in the comprehensive evaluation model for soil. Table 4 showed the coordination relationship between vegetation and soil under different years of restoration. The comprehensive evaluation indices of vegetation growth in the 10th and 15th years of restoration were both 0.79, while those of soil quality were 0.81 and 0.80, respectively. The vegetation‐soil systems in the unrestored period and the 1st year of restoration were in the stage of intermediate coordinated development, whereas those in the 5th, 10th, and 15th years of restoration were in the stage of well‐coordinated development. Specifically, the vegetation‐soil coupling coordination degree reached the maximum value of 0.634 in the 10th year of restoration. The comprehensive coordination index in the late stage was greater than that in the unrestored stage and initial stage, and peaked in the 10th year of restoration. The comprehensive evaluation index of vegetation growth was significantly affected by vegetation coverage, plant leaf and root nutrient contents, and plant diversity indices, while that of soil quality was affected by soil physicochemical properties, soil depth, and restoration years (Figure 7).

**TABLE 4:** Evaluation results of vegetation–soil coordination relationship.

Year	VCE	SCE	C	Ch	Cd	Coupling mode
0	0.653	0.746	0.500	0.700	0.591	Medium level coordinated development model
1	0.657	0.763	0.500	0.710	0.595	Medium level coordinated development model
5	0.763	0.775	0500	0.769	0.620	Good coordinated development model
10	0.794	0.813	0.500	0.803	0.634	Good coordinated development model
15	0.793	0.802	0.500	0.798	0.631	Good coordinated development model

**FIGURE 8:**
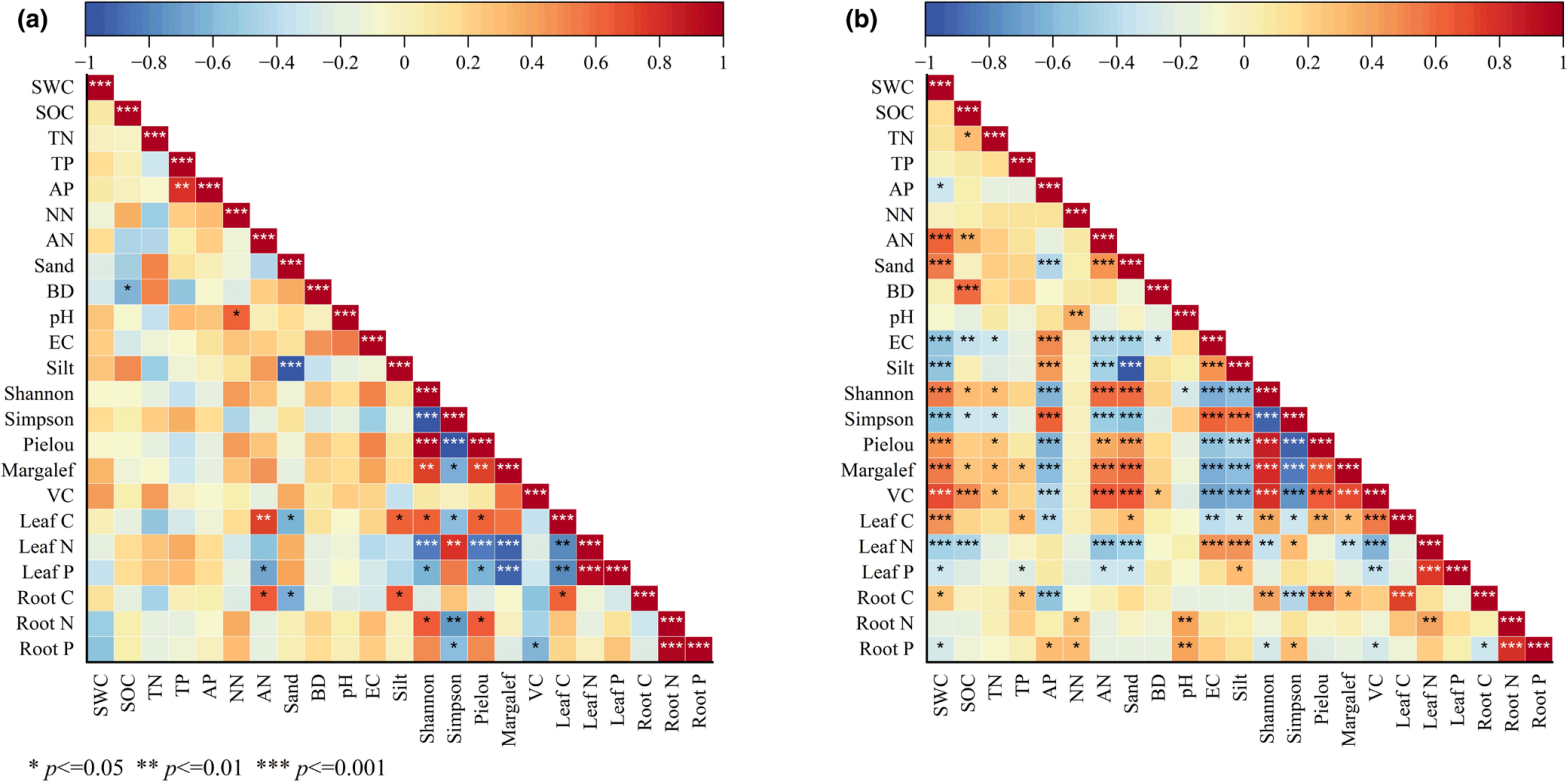
Correlation between vegetation and soil properties during unrestored and restored stages. (a) unrestored stages (year 0); (b) restored stages.

As shown in Figure 8, there existed varying degrees of correlation between vegetation characteristics and soil properties among restored and unrestored stages. The correlation between vegetation and soil in unrestored stages was markedly weaker than that in restored stages. Under artificial restoration intervention, plant diversity, soil nutrient contents, vegetation coverage, and plant leaf and root nutrient contents all showed significant correlations. Conversely, in unrestored stages, soil physical properties such as bulk density, pH, and electrical conductivity (EC) showed no apparent correlation with plant characteristics. However, this correlation became significantly stronger in restored stages. Similarly, the correlation between plant nutrient contents and soil nutrient contents demonstrated increased correlation due to artificial restoration efforts.

## 4 Discussion

### 4.1 Effect of Restoration Year on Vegetation Properties

**FIGURE 9:**
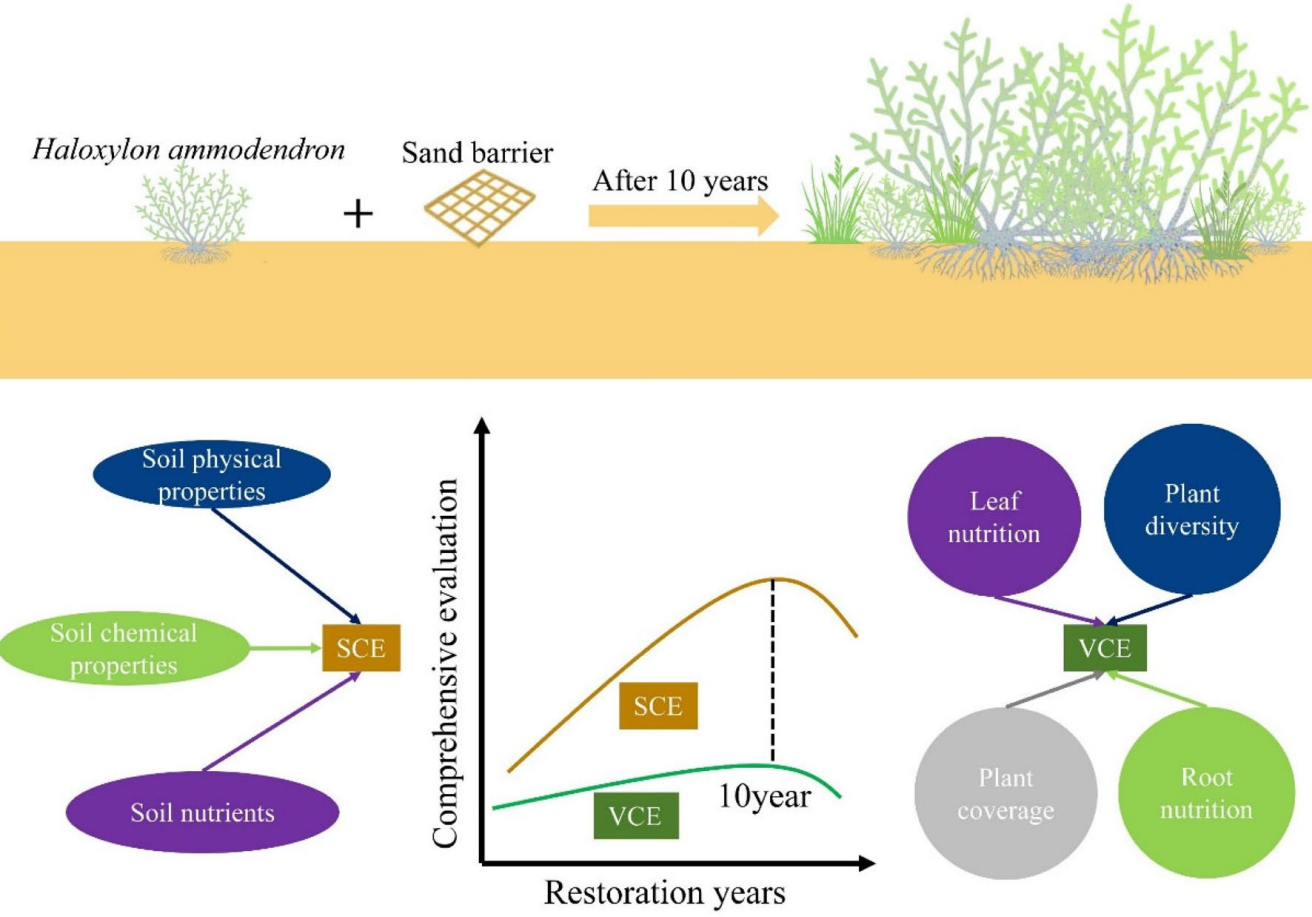
Schematic diagram of vegetation–soil system succession under artificial sand control.

Vegetation restoration is the most effective way to control soil erosion in the Ulan Buh Desert, given its long‐term effect on the degraded ecosystem. The coupling coordination between vegetation and soil is crucial to the successful implementation of ecological restoration and the Three‐North Shelterbelt Project. However, little is known about the dynamics of vegetation and soil properties under different years of restoration, such as whether soil fertility is improved year by year, to what extent plant community diversity and stability will recover under long‐term restoration efforts, and whether there exists a threshold in the restoration processes. The results showed that plant species number increased and then stabilized as restoration progressed, and peaked in the 15th year of restoration, when the plant community was mainly composed of Chenopodiaceae, *Poaceae*, and *Asteraceae* species (Tables 1 and 2). *Agriophyllum* *pungens* and *Phragmites australis* were pioneer species in the early stages of restoration. Species such as *Grubovia* *dasyphylla*, *Salsola collina* , and *Artemisia desertorum* appeared after *Haloxylon ammodendron* was introduced for wind break and sand fixation. Plant community succession tended to stabilize when *Artemisia desertorum and Haloxylon ammodendron* matured and sand control achieved initial success. A previous study has shown that *Chenopodiaceae, Fabaceae, and Poaceae* plants would appear after 10 years of restoration in desertified areas, while those unrestored areas only have a monotonous species composition (1 or 2 species) (Hu et al. 2016). Given the peculiarity of desertified areas, this study mainly investigated shrub and herb species in the Ulan Buh Desert. Undoubtedly, *Haloxylon ammodendron*, as a typical desert plant, has an ability to prevent soil erosion. As illustrated in Figure 9, the introduction of *Haloxylon ammodendron* and sand barriers drives a clear succession of the vegetation‐soil system, with soil comprehensive evaluation (SCE) and vegetation comprehensive evaluation (VCE) showing distinct dynamic patterns over restoration years. Nevertheless, further research is needed to explore the characteristics of plants surrounding *Haloxylon ammodendron* and the mechanisms of plant diversity dynamics with the progress of vegetation restoration.

Vegetation restoration can effectively improve plant leaf and root traits, such as nutrient content (Chen et al. 2023). In the present study, leaf nutrient content showed notable shifts across different years of restoration (Figure 3), revealing the nutrient adaptation strategies of vegetation in arid regions. Leaf total nitrogen content reached the maximum value in the 10th year of restoration and decreased in the 15th year, which is consistent with the findings in karst rocky desertified areas that leaf total nitrogen content increased and then decreased with the progress of long‐term vegetation restoration (Hu et al. 2023). In addition, we observed that leaf organic carbon content decreased and then increased during the initial phase of vegetation restoration and tended to stabilize after 10 years of restoration. Such “carbon fluctuation” phenomenon can be explained by the carbon allocation theory in ecological stoichiometry: when vegetation shows a succession from annual herbs to perennial shrubs, the proportion of carbon allocated to roots increases from 30% to 55%, resulting in a relative decrease in leaf organic carbon concentration (Aerts and Chapin 2000). It reflects that vegetation in arid regions relies more on inter‐organ nutrient reallocation than internal homeostasis regulation to adapt to nutrient fluctuations (Wang et al. 2023).

Plant roots play a beneficial role in the material cycle and energy flow in ecosystems, which further enhances ecosystem services (Williams and De Vries 2020). Our results showed that root nutrient content in the late stage of restoration was significantly higher than that in the unrestored stage and initial stage (*p* < 0.05), which is in agreement with the findings of a previous study (Xu et al. 2021).

### 4.2 Effect of Restoration Year on Soil Properties

We found that soil physicochemical properties differed significantly among different years of restoration, aligning with a previous study on the influence of ecological restoration on soil physical properties in arid areas (Wang and Wang 2019). Soil physical properties such as bulk density and soil moisture were better improved in the late stage of restoration compared to the unrestored stage and initial stage. The average soil bulk density of the 0–20 cm soil layer in the unrestored stage was 11.54% higher than that in the 15th year of restoration. The average soil moisture of each soil layer in the 15th year of restoration was increased by 50.00%–76.67%, compared to the unrestored stage. Soil structure arrangement determines soil particle size distribution (Qiu et al. 2022). Soil particle size is a highly dynamic attribute which is influenced by various natural and artificial factors. As a result, understanding its temporal variability is a basic requirement for the accurate description of soil dynamics in the restoration processes (Uteau et al. 2013). We found that long‐term restoration significantly decreased sand content and increased silt content, suggesting that vegetation restoration gradually improves the texture of sandy soil by alleviating wind erosion and enhancing fine particle deposition. This improvement in soil texture, together with the continuous reduction of surface soil bulk density, directly optimizes the soil pore structure—enlarging the proportion of small and medium pores that are critical for water retention, and thus significantly enhancing the long‐term water‐holding capacity of the sand‐fixing area. The stable and high soil moisture content in the 10th year of restoration further confirms that the soil water retention system has matured and stabilized at this stage, which is a key hydrological characteristic for maintaining the stability of desert vegetation communities and resisting drought stress in arid regions. These optimization measures not only restructured soil pore system but also provided a physical basis for subsequent ecological processes including water retention and nutrient cycles, highlighting the critical effect of soil texture improvement in the restoration of desert ecosystems.

Moreover, changes in soil chemical properties can reflect the shifts in soil fertility during restoration. Soil pH showed no significant differences across different years of restoration and soil depths, but gradually decreased over time, which may be attributed to the accumulated organic acids derived from the decomposition of root exudates and plant litter (Zhou et al. 2023). Despite being a subtle shift, it may slowly alter soil pH and facilitate nutrient release. In contrast, soil EC differed markedly across different years of restoration (*p* < 0.001) and was apparently lower in the late stage, indicating that long‐term restoration reduces soil salinity, which is favorable for plant growth. This finding is consistent with the case that increased vegetation coverage would promote salt leaching in deserts (Sudduth et al. 2005).

Soil organic carbon (SOC) increased in a wave‐like pattern. SOC of the surface and deep soil layers peaked in the 15th year of restoration, while that of the middle soil layer peaked in the 10th year. This phenomenon is likely related to litter input dynamics and root distribution in different soil layers, as deeper shrub roots in the late stage may transport more carbon to deep soils (Zhou et al. 2018). Total nitrogen (TN) content varied between soil depths. Specifically, TN content of the surface and deep soil layers peaked in the 10th year of restoration, while that of the middle soil layer peaked in the 15th year. In addition, the synergistic effect of restoration year and soil layer had an enormous impact on soil TN content. These results are likely due to herb‐to‐shrub succession and varying microbial activities across soil layers (Wang et al. 2022). Soil available nitrogen (AN) was highest in the late stage, peaking in the 10th year of restoration across all soil layers, probably because root exudates have enhanced microbial nitrogen mineralization in this period (Chen et al. 2023). Soil total phosphorus (TP) differed significantly across different years of restoration. Specifically, soil TP content of the surface and deep soil layers respectively peaked in the 5th and 10th year of restoration, while that of the middle soil layer peaked before the onset of restoration. Available phosphorus (AP) and nitrate nitrogen (NN) respectively changed 0.02–0.04 mg/g and 0.004–0.005 mg/g on average across restoration periods, and thus did not show any significant shifts. Overall, soil chemical properties were greatly improved and tended to stabilize after 10 years of restoration, which aligns with the enhancement in soil physical properties. Increased silt content led to a more optimal pore structure which further facilitated nutrient retention and microbial activity, while the accumulation of soil nutrients in turn enhanced soil structure. The interactions of both processes jointly boosted soil fertility.

Our results showed that during the same restoration period, soil physicochemical properties displayed significant differences across soil layers (*p* < 0.05). This spatial heterogeneity was driven by the synergistic effect of restoration method and soil depth (Jiang et al. 2019). The effect of vegetation restoration on soil texture (Wu et al. 2016) was significantly concentrated in the surface soil (0–10 cm, especially 0–5 cm), and exhibited an exponential decay with increasing soil depth, which is in accordance with the result of previous studies (Li et al. 2018). Meanwhile, this observation is highly consistent with our findings that physicochemical properties of deep soils rarely showed significant differences across different years of restoration. The improvement of surface soil quality primarily results from the ecological regulation of vegetation, including the organic matter layer formed by the humification of plant litter, the physical restructuring of soil structure through root networks, and biochemical enhancement of surface soil triggered by rhizosphere exudates (Bai et al. 2016; Zhang et al. 2019). Compared with surface soil, deep soil displayed a significantly delayed restoration process of soil physicochemical properties, probably attributed to a weakened direct influence of plant physiological activities on it. This differentiation not only reflects the dependence and limitation of ecological restoration in arid regions, but provides new insight into the formulation of restoration strategies that specific targeted measures should be taken to restore different soil layers.

### 4.3 Response of Vegetation‐Soil Coupling Relationship to Restoration Year

As shown in Figure 8, there existed different degrees of correlations between vegetation characteristics and soil properties across different years of restoration. Due to artificial restoration interventions, there was a strong correlation between vegetation diversity, root characteristics, and soil physical properties. However, in unrestored mobile dune areas, this correlation was generally weak. Such dynamic changes primarily resulted from the mutual feedback within the vegetation‐soil system (Li et al. 2018). Vegetation restoration gradually improves habitat soil conditions, while the improved soil conditions in turn regulate the development process of vegetation (Wang and Wang 2019). The impact of these interactions on ecological restoration can be either positive or negative, depending on the coordinated development between vegetation and soil (L. Feng et al. 2023; R. Gao et al. 2022; T. Hu et al. 2023). This study revealed that both Vegetation Condition Index (VCE) and Soil Condition Index (SCE) were significantly higher during various stages of restoration (1, 5, 10, and 15 years) than those observed during the unrestored stage. Due to natural succession of plant communities, VCE was relatively high after 10 years of restoration, indicating a good condition of vegetation. Meanwhile, the increased SCE also indicated a positive effect of restoration on soil conditions. However, different restoration stages are faced with distinct challenges. During the unrestored and initial stages (1 year), there is a low level of soil nutrients and vegetation coverage, and the overall ecosystem functioning is weak. During the middle stage (5 years), soil nutrient levels and vegetation coverage have been significant enhanced, but plant species composition remains relatively homogeneous with a low diversity level. During the late stage (10–15 years), there is a high level of vegetation coverage and species diversity, but soil nutrient content emerges as a new limiting factor. Therefore, phased and targeted management strategies should be adopted to achieve effective ecological restoration. During the initial and middle stages (< 10 years), focus should be placed on soil conservation (e.g., erosion reduction) and precise nutrient management (e.g., appropriate fertilization) to support plant establishment and early growth. During the late stage (≥ 10 years), management efforts should be shifted to maintaining or enhancing plant diversity, optimizing species composition, and improving ecosystem complexity and stability.

## 5 Conclusions

This study focused on the ecological restoration areas under different years of restoration in the Ulan Buh Desert, analyzing the vegetation characteristics, plant nutrient contents, soil physicochemical properties, and the coordinated development of the vegetation‐soil system in these areas. The results showed that there were significant differences in vegetation characteristics and soil physicochemical properties among different years of restoration. The colonization and community development of desert plants such as *Artemisia desertorum* played a key role in the restoration of ecosystem structure and function, especially sand fixation function.

The comprehensive evaluation model showed that the ecological restoration project achieved initial success after 10 years of efforts. The coupling degree and coupling coordination degree model demonstrated that the vegetation‐soil system entered the stage of well‐coordinated development after 10 or more years of restoration. These findings will provide an important scientific basis for late‐stage management of sand control.

## Author Contributions

**Benmo Li:** data curation (equal), formal analysis (equal), methodology (equal), writing – original draft (lead). **Dong Wang:** resources (equal). **Yujie Xue:** software (lead). **Runze Liu:** visualization (equal). **Jingwen Li:** writing – review and editing (equal).

## Funding

This work was supported by the China State Construction Engineering Corporation (CSCEC) under the project Research and Application of Key Technologies for Ecological Environment Protection and Restoration in the Northwest Region (CSCEC‐2020‐Z‐5), and the National Natural Science Foundation of China (32271703).

## Conflicts of Interest

The authors declare no conflicts of interest.

## Data Availability

The main datasets supporting the analyses are available on FigShare at https://doi.org/10.6084/m9.figshare.31697956.
